# Rituximab Therapy for Immune-Mediated Neurological Diseases: Our Experience at a Tertiary Care Centre

**DOI:** 10.7759/cureus.62227

**Published:** 2024-06-12

**Authors:** Pranit D Khandait, Shalesh Rohatgi, Satish P Nirhale, Prajwal M Rao, Pravin U Naphade

**Affiliations:** 1 Neurology, Dr. D. Y. Patil Medical College, Hospital & Research Centre, Dr. D. Y. Patil Vidyapeeth (Deemed to be University), Pune, IND

**Keywords:** immune-mediated neurological diseases, nmosd, mogad, ms, anti-cd-20, rituximab

## Abstract

Introduction: Rituximab (RTX) is a monoclonal anti-CD20 chimeric antibody that inhibits B cell activity. However, it is an appealing substitute for traditional immunomodulatory drugs as a swiftly acting, targeted therapy with mounting evidence of efficacy and tolerance in numerous neuroinflammatory conditions. We discuss the scientific evidence for the use of RTX in neurological illnesses, as well as the dose, safety, and other practical elements of prescription.

Aim: This study aims to assess and correlate the effects of RTX on immune-mediated neurological disorders.

Objectives: The primary objective of this study is to determine the outcomes in patients treated with RTX for the following conditions: myasthenia gravis (MG), autoimmune encephalitis, multiple sclerosis (MS), neuromyelitis optica spectrum disorder (NMOSD), myelin oligodendrocyte glycoprotein antibody disease (MOGAD), immune-mediated peripheral neuropathy, and inflammatory muscle disease. The secondary objective is to assess adverse drug reactions in patients treated with RTX.

Methods: This is a prospective observational study conducted at a tertiary care centre. The data were analyzed for the period from May 2022 to May 2024. Approval was obtained from the institutional ethics committee before commencing the study, and written informed consent was obtained from all patients.

Results and conclusions: A total of 56 patients were included in the study. The distribution of patients according to diseases is as follows: MG (17), MS (11), NMOSD (10), MOGAD (7), immune-mediated peripheral neuropathy (6), autoimmune encephalitis (3), and inflammatory muscle disease (2). However, one patient was lost to follow-up in the autoimmune encephalitis group. All patients experienced improvements in symptoms, and no relapse episodes have been reported except for one patient who had a relapse in the inflammatory muscle disease group. During the infusion process, some adverse drug reactions, such as chills and rigors, were observed, and two patients experienced major side effects, such as Pott's disease and cryptogenic organizing pneumonia. Nevertheless, overall, rituximab shows promise as an off-label immunosuppressive treatment for the aforementioned neurological immune-mediated diseases.

## Introduction

Rituximab (RTX) is a chimeric antibody that acts as an inhibitor of B cells by targeting CD20 [1,2]. In 1980, RTX was first administered to a patient with lymphoma by Lee Nadler at the Dana-Farber Cancer Institute using the Köhler-Milstein technique [3,4]. Antibodies can be classified into various immunoglobulin classes (M, G, D, E, or A) and subclasses (such as IgG1-4). Additionally, these antibodies are linked with immune-mediated neurological diseases [5]. For the past 11 years, RTX has been extensively utilized as an off-label therapy for individuals with multiple sclerosis (MS) and has shown reduced relapse rates and disability progression in MS. Additionally, RTX has been used off-label for other immune-mediated neurological diseases mentioned above.

Beneficial outcomes have been documented in terms of reducing relapses and decreasing the development of the disease. Crucially, a sizable observational study of Swedish MS patients revealed no significant side effects from its sustained use [6]. Many other trials from different nations have validated the safety and effectiveness of RTX in lowering relapses and disability in neuromyelitis optica spectrum disorder (NMOSD) since Cree et al.'s initial report on the drug's effectiveness in treating the illness [7-9]. RTX has also shown promising outcomes in cases of myelin oligodendrocyte glycoprotein antibody disease (MOGAD) and immune-mediated peripheral neuropathy [10-12]. Additionally, RTX has demonstrated promising outcomes in the treatment of autoimmune encephalitis (AE) and inflammatory muscle disease.

## Materials and methods

We conducted a prospective observational study on patients with immune-mediated neurological diseases who presented at a tertiary care hospital in India from May 2022 to May 2024. Approval was obtained from the institutional ethics committee of Dr. D. Y. Patil Medical College, Hospital & Research Centre (IESC/S.SP/01/2022) before commencing the study, and written informed consent was obtained from all patients. The sample size was calculated using the method described for a descriptive prevalence study based on effect size and absolute precision. The minimum sample size for the study was determined to be 30 subjects based on the prevalence of the disease mentioned in prior studies. A total of 56 patients were included in this study.

Inclusion criteria

The study included patients with the following immune-mediated neurological diseases: (1) myasthenia gravis (MG), MS, NMOSD, MOGAD, immune-mediated peripheral neuropathy, inflammatory muscle disease, and AE; (2) all patients willing to give informed consent for their participation in the study; and (3) age more than 18 years old.

Exclusion criteria

Excluded in the study are (1) patients who are not willing to give consent for participation in the study; (2) pregnant women in the first trimester (RTX is relatively safe during pregnancy; however, the first trimester is a critical phase of organogenesis, and there are a few reports that suggest it may cause abortion if used in the first trimester. Complications observed in the second and third trimesters are very rare and manageable); and (3) patients having active infections such as tuberculosis, progressive multifocal leukoencephalopathy, HIV, hepatitis B surface antigen (HbsAg), or hepatitis C virus (HCV) (all chronic infections) were considered in order to prevent a flare-up of such infections.

Relapse was defined by new neurological symptoms or worsening of existing symptoms, which may be associated with worsening observed in laboratory or radiological assessments. Even a mild worsening of symptoms was considered. For example, in the case of MG, an increase in fatigability or worsening in the MGFA (MG-Foundation of America) class or other scales mentioned below; in the case of MS/NMOSD/MOGAD, new neurological symptoms or worsening of existing symptoms associated with radiological worsening; and in the case of other immune-mediated neurological diseases, we monitored them closely using respective scales/scores and also monitored laboratory or radiological parameters. In the event of a relapse, an alternative treatment therapy was considered.

The protocol for RTX infusion at our centre

Written informed consent was obtained from the patients, and the benefits and complications regarding RTX were explained to the patient and their relatives. On the day of RTX infusion, it was important to clinically rule out active infection. Routine blood investigations, including a complete blood count, renal and liver function tests, sero-markers (HIV/HbsAg/HCV), urine routine microscopy, and chest X-ray, were performed.

Before starting RTX infusion, premedication with intravenous 125 mg methylprednisolone and intravenous pantoprazole 40 mg was administered. RTX was slowly administered with an initial infusion rate of 10 mL/hour, increasing every 10 minutes to 150 mL/hour, with cardiac monitoring. A total of 1 g was given in two divided doses, each in 500 mL of normal saline. The induction dose of 1 g RTX was administered 15 days apart, followed by maintenance doses every six months.

For female patients of reproductive age, counseling regarding conception and the risks and benefits of RTX was provided. We explained to patients the importance of planning their pregnancies accordingly and advised them on the timing of RTX doses prior to and just after delivery or in the second or third trimester.

Patient outcomes assessed with various parameters

For MG, outcomes were assessed using the following tools: Quantitative-MG (QMG), MGFA scale, MG-Activities of Daily Living (MG-ADL), MG-Quality of Life-15-revised (MG-QoL-15r), and MG-Composite Scale (MG-CS). For NMOSD, MOGAD, and MS, the Expanded Disability Status Scale (EDSS), Radiological lesions per year, and Fatigability Severity Scale (FSS) were used. For immune-mediated peripheral neuropathy, the Modified Neuropathy Symptoms Score stage (MNSS), FSS, Neuropathy Disability Score (NDS), and Shortened and Revised Total Neuropathy Scoring (SRTNS) were employed. For AE, the Clinical Assessment Scale in AE (CASE), Radiological lesions per year, and Modified Ranking Scale (MRS) were utilized. For inflammatory muscle disease, the creatine phosphokinase-N-acetyl cysteine (CPK-NAC) levels were used. The annual relapse rate (ARR) was considered in all the above conditions.

Data were collected from hospital admission records. Follow-up clinical comparative examinations and improvement assessments were conducted every six months until the last follow-up, with additional assessments as needed. MRI scans were performed yearly to detect new lesions. Outcomes were analyzed based on demographic details and the number of doses of RTX, as well as its efficacy in the form of clinical scales, laboratory, radiological improvement, and ARR related to the disease. Safety was assessed in terms of drug infusion-related reactions, as well as consideration of long-term infections or reactivation of infections.

Statistical analysis

Parametric variables, representing normally distributed data, were depicted using both the mean and median. Data were compared from the baseline clinical scales, laboratory results, and radiological patient data to the most recent follow-up.

## Results

The study comprised 56 patients, of whom 24 were males and 32 were females. The males had an average age of 49, whereas the females had an average age of 37 (Table 1).

**Table 1:** Demographic data of the patients who received rituximab. NMO/NMOSD: neuromyelitis optica/neuromyelitis optica spectrum disorder; MOGAD: myelin oligodendrocyte glycoprotein antibody disease.

Disease	Frequency of Patients in a Study	Percentage (% Distribution of Patients in a Study)	Mean Age (Year)	M:F
Myasthenia gravis	17	30	53.2 ± 17.6	1.4:1
Multiple sclerosis	11	20	29.6 ± 9.9	1:1.7
NMO/NMOSD	10	18	34.5 ± 13.3	1:9
MOGAD	7	12	38.3 ± 18.9	1:1.3
Immune-mediated peripheral neuropathy	6	11	54.3 ± 17.7	2:1
Autoimmune encephalitis	3	5	42.3 ± 21.7	2:1
Inflammatory muscle disease	2	4	51.0 ± 17.0	0:2
Total patients	56 (male-24, female-32)	100	43 (male-49, female-37)	1:1.3

Patients with MG

A total of 17 individuals were diagnosed with MG (10 males and 7 females). Baseline to follow-up comparisons were made for MGFA class, MG-CS, MG-ADL, MG-Qol-15r, and QMG scores that showed significant improvements with an ARR of 0 (Table 2).

**Table 2:** Efficacy of rituximab in a group of patients with MG. MG: myasthenia gravis; AChRa: acetylcholine receptor; MUSK: muscle-specific kinase; MGFA: MG-Foundation of America; MG-CS: MG-Composite Scale; MG-ADL: MG-Activities of Daily Living; MG-QoL-15r: MG-Quality of Life-15-revised; QMG: Quantitative-MG; NA: not applicable.

Myasthenia Gravis (N=17)				
Age (in years)	53.2 ± 17.6			
Female; N (%)	7 (41%)			
AChRa-positive myasthenia gravis	13 (76%)			
MUSK-positive myasthenia gravis	3 (18%)			
MUSK-positive, anti-striatal muscle antibody-positive myasthenia gravis	1 (6%)			
	At presentation (N=17)	At 6 months (N=14)	At 12 months (N=8)	At 18 months (N=2)
MGFA class				
I	03	00	00	00
II	05	00	00	00
IIA	01	00	00	00
IIB	03	00	00	00
III	02	00	00	00
IIIB	01	00	00	00
IVB	01	00	00	00
V	01	00	00	00
MG scales				
MG-CS	13.5 ± 11.9	0	0	0
MG-ADL	7.6 ± 4.9	0	0	0
MG-QoL-15r	12.4 ± 6.4	0	0	0
QMG	10.8 ± 7.6	0	0	0
Relapse episode/year	NA	NA	0	0

Patients with NMOSD

**Figure 1:**
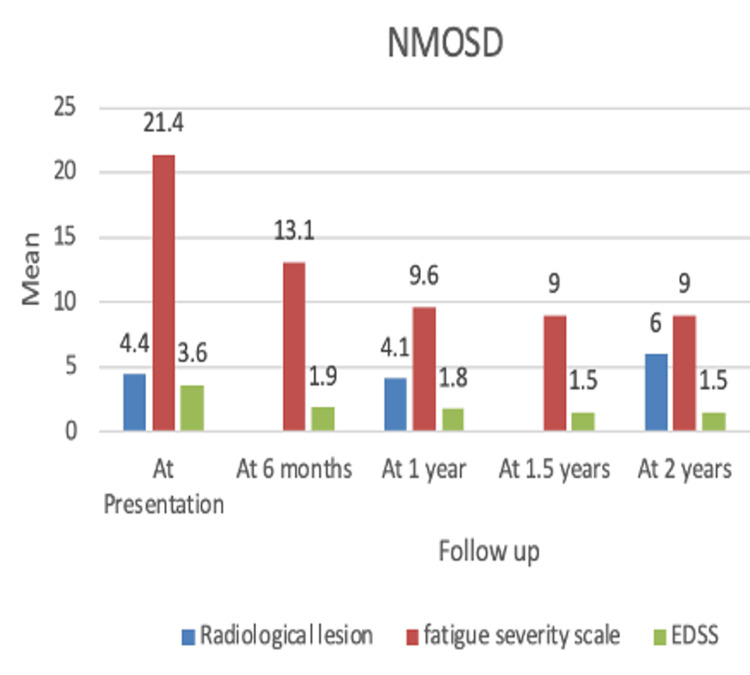
Bar diagram showing the efficacy of rituximab in a group of patients with NMOSD. NMOSD: neuromyelitis optica spectrum disorder.

A total of 10 patients diagnosed with NMOSD underwent RTX treatment, comprising nine females and one male. Radiological lesions at presentation had a mean ± SD and median (IQR) of 4.4 ± 4.0 and 2.5 (2-6.75), respectively, (n=10). At one year (n=8), it was 4.1 ± 4.4 and 2 (0.75-8.25); at two years (n=3), it was 6.0 ± 4.4 and 8 (4.5-8.5). One patient who completed two years of the study had a baseline lesion load of 11, which reduced to nine at two years. Overall, radiological lesions in the above population did not increase, and no new enhancing lesions were observed, showing a significant response to treatment. EDSS showed significant improvement from 3.6 ± 1.3 to 1.5 ± 1.3 (Table 3) (Figure 1). All patients were in the remission phase (ARR=0).

**Table 3:** Efficacy of RTX in a group of patients with NMOSD. NMOSD: neuromyelitis optica spectrum disorder; EDSS: Expanded Disability Status Scale; IQR: interquartile range; NA: not applicable.

Neuromyelitis Optica Spectrum Disorder (NMOSD) (N=10)					
Age (in years)	34.5 ± 13.3				
Female; N (%)	9 (90%)				
	At Presentation (N=10)	At 6 months (N=9)	At 12 months (N=8)	At 18 months (N=5)	At 24 months (N=3)
Radiological lesion (mean ± SD; median (IQR))	4.4 ± 4.0; 2.5 (2-6.75)	NA	4.1 ± 4.4; 2 (0.75-8.25)	NA	6.0 ± 4.4; 8 (4.5-8.5)
Fatigue severity scale	21.4 ± 13.4	13.1 ± 10.2	9.6 ± 1.8	9.0 ± 0.0	9.0 ± 0.0
EDSS	3.6 ± 1.3	1.9 ± 1.6	1.8 ± 1.5	1.5 ± 1.4	1.5 ± 1.3
Relapse episode/year	NA	NA	00	00	00

Patients with MOGAD

**Figure 2:**
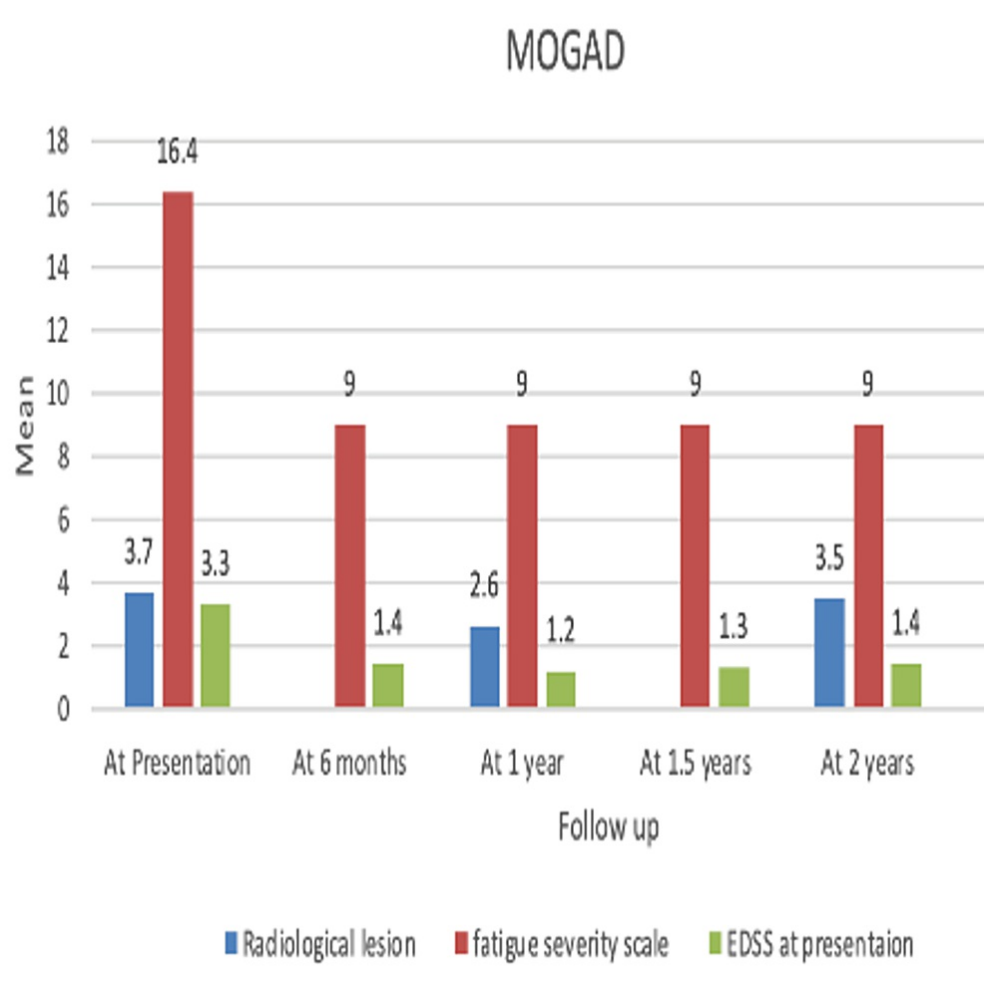
Bar diagram showing the efficacy of rituximab in a group of patients with MOGAD. MOGAD: myelin oligodendrocyte glycoprotein antibody disease; EDSS: Expanded Disability Status Scale.

Seven patients diagnosed with MOGAD underwent RTX treatment, with an F:M ratio of 1.3:1 and an average age of 38.3 (±18.9). Radiological lesions at presentation had a mean ± SD and median (IQR) of 3.7 ± 4.7 and 2 (1-3.5), respectively, (n=7). At one year (n=7), it was 4.1 ± 4.4 and 1 (0.75-8.25); at two years (n=4), it was 3.5 ± 5.7 and 1 (0.75-3.75). One patient who completed two years of the study had a baseline lesion load of 14, which reduced to 12 at two years. Overall, radiological lesions in the above population did not increase, and no new enhancing lesions were observed, showing a significant response to treatment. Comparing FSS and EDSS showed significant improvement (Table 4) (Figure 2). All patients were in the remission phase (ARR=0).

**Table 4:** Efficacy of RTX in a group of patients with MOGAD. MOGAD: myelin oligodendrocyte glycoprotein antibody-associated disease; IQR: interquartile range; EDSS: Expanded Disability Status Scale; NA: not applicable.

Myelin Oligodendrocyte Glycoprotein Antibody-Associated Disease (N=7)					
Age (in years)	38.3 ± 18.9				
Female; N (%)	4 (57%)				
Patients follow-up	At presentation (N=7)	At 6 months (N=7)	At 24 months (N=7)	At 18 months (N=6)	At 24 months (N=4)
Radiological lesion/year (mean ± SD; median (IQR))	3.7 ± 4.7; 2 (1-3.5)	NA	2.6 ± 4.3; 1 (0.5-2)	NA	3.5 ± 5.7; 1 (0.75-3.75)
Fatigue severity scale	16.4 ± 5.0	9.0 ± 0.0	9.0 ± 0.0	9.0 ± 0.0	9.0 ± 0.0
EDSS at presentation	3.3 ± 0.4	1.4 ± 1.3	1.2 ± 1.1	1.3 ± 1.1	1.4 ± 1.1
Relapse episode/year	NA	NA	00	00	00

Patients with MS

**Figure 3:**
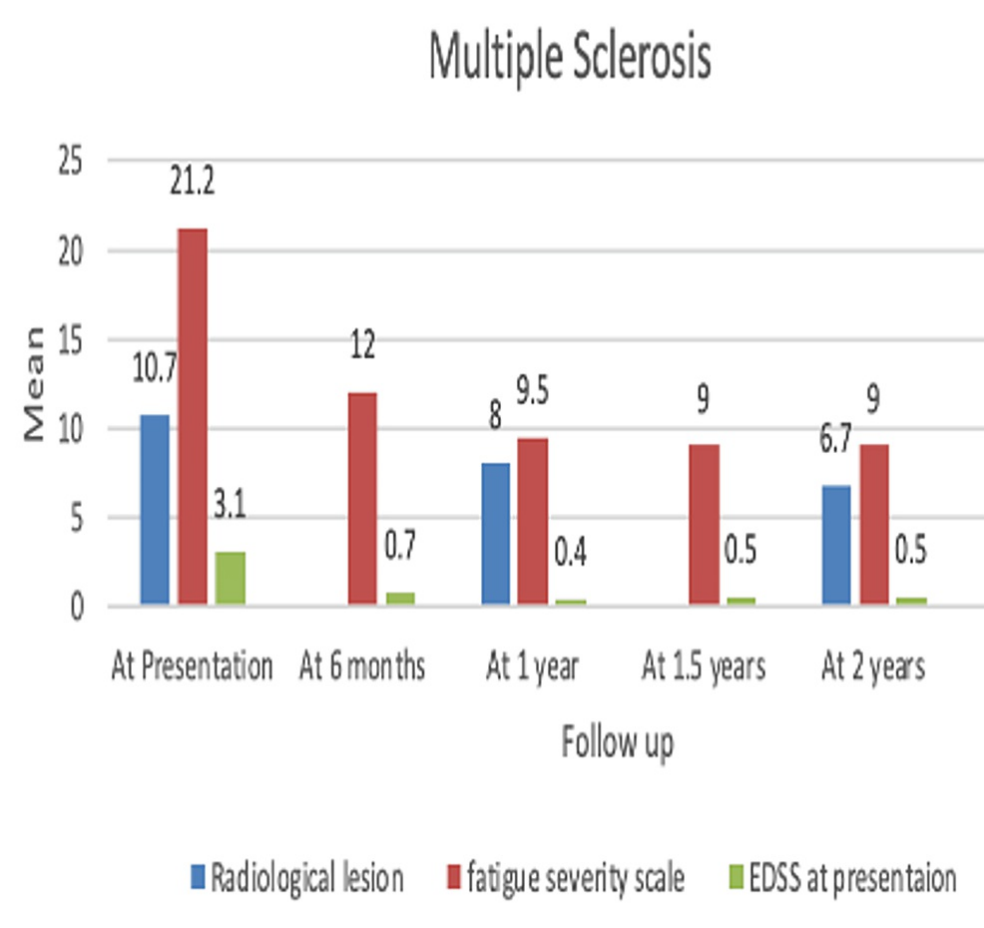
Bar diagram showing the efficacy of rituximab in a group of patients with MS. MS: multiple sclerosis; EDSS: Expanded Disability Status Scale.

A total of 11 patients diagnosed with MS underwent RTX treatment (F:M=1.75:1), with an average age of 29.6 (±9.9). Radiological lesions at presentation had a mean ± SD of 10.7 ± 5.6 (n=11). At one year (n=7), it was 8.0 ± 2.8, and at 2 years (n=4) it was 6.7 ± 2.3. Overall, radiological lesions in the above population did not increase, and no new enhancing lesions were observed, showing a significant response to treatment. Comparing EDSS at baseline showed a mean ± SD of 3.1 ± 1.3 (n=11). At one year (n=7), it was 0.4 ± 0.7, and at two years (n=4) and 2.5 (n=1) years, it was 0.5 ± 0.9, showing significant improvement (Table 5) (Figure 3). All patients were in the remission phase (ARR=0).

**Table 5:** Efficacy of rituximab in a group of patients with MS. MS: multiple sclerosis; EDSS: Expanded Disability Status Scale; NA: not applicable.

Multiple Sclerosis (N = 11)					
Age (in years)	29.6 ± 9.9				
Female; N (%)	7 (63.6%)				
Patients follow-up	At presentation (N=11)	At 6 months (N=9)	At 12 months (N=7)	At 18 months (N=6)	At 24 months (N=4)
Radiological lesion/year	10.7 ± 5.6	NA	8.0 ± 2.8	NA	6.7 ± 2.3
Fatigue severity scale	21.2 ± 8.8	12.0 ± 3.6	9.5 ± 1.2	9.0 ± 0.0	9.0 ± 0.0
EDSS at presentation	3.1 ± 1.3	0.7 ± 0.9	0.4 ± 0.7	0.5 ± 0.7	0.5 ± 0.9
Relapse episode/year	NA	NA	00	00	00

Patients with IMPN

**Figure 4:**
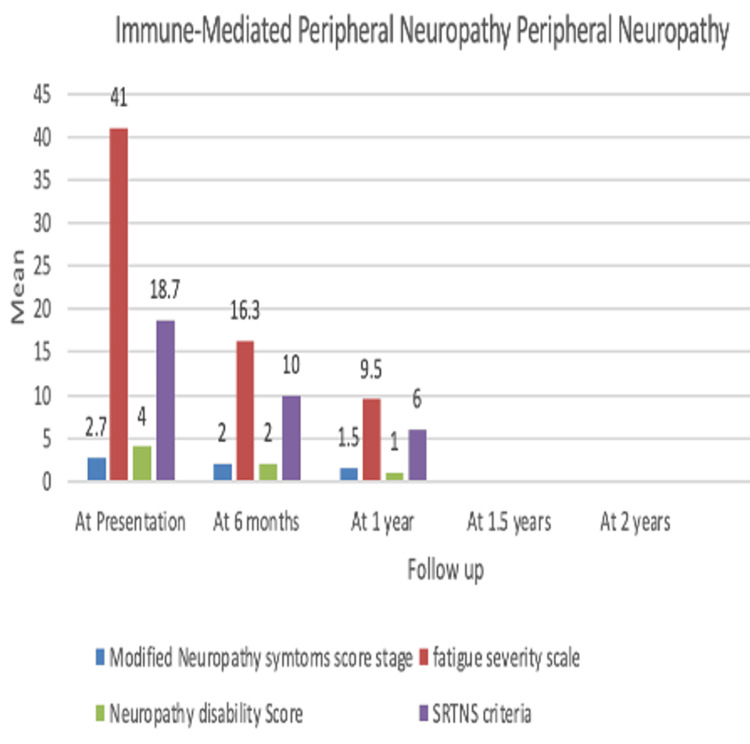
Bar diagram showing the efficacy of rituximab in a group of patients with IMPN. IMPN: immune-mediated peripheral neuropathy; SRTNS: Shortened and Revised Total Neuropathy Scoring.

Six individuals diagnosed with IMPN underwent treatment with RTX, among whom three (66%) had chronic inflammatory demyelinating polyneuropathy (CIDP) (with two patients experiencing relapse on azathioprine), one (33%) had nodo-paranodopathy, and one (33%) had seronegative systemic vasculitic neuropathy. This group comprised an M:F ratio of 2:1, with an average age of 54.3 (±17.7). Comparing MNSS, FSS, NDS, and SRTNS criteria from baseline to follow-up showed significant improvement (Table 6) (Figure 4). All patients were in the remission phase (ARR=0).

**Table 6:** Efficacy of rituximab in a group of patients with IMPN. IMPN: immune-mediated peripheral neuropathy; CIDP: chronic inflammatory demyelinating polyneuropathy; SRTNS: Shortened and Revised Total Neuropathy Scoring; NA: not applicable.

Immune-Mediated Peripheral Neuropathy (N=6)			
Age (in years)	54.3 ± 17.7		
Female; N (%)	2 (33.3%)		
CIDP	3 (66.7%)		
Nodopathy paranodopathy	1 (16.7%)		
Seronegative systemic vasculitis neuropathy	1 (16.7%)		
Patients follow-up	At presentation (N=6)	At 6 months (N=4)	At 12 months (N=2)
Modified neuropathy symptoms score stage	2.7 ± 0.5	2.0 ± 0.8	1.5 ± 0.7
Fatigue severity scale	41.0 ± 16.6	16.3 ± 5.5	9.5 ± 0.7
Neuropathy disability score	4.0 ± 1.1	2.0 ± 1.4	1.0 ± 1.4
SRTNS criteria	18.7 ± 2.0	10.0 ± 7.2	6.0 ± 8.5
Relapse episode/year	NA	NA	0

Patients with AE

Three patients diagnosed with AE underwent rituximab treatment, out of which one (33.33%) had glutamic acid decarboxylase 65-kilodalton (GAD-65) antibody, one (33.33%) had N-methyl-D-aspartate receptor (NMDAR) antibody, and one (33.33%) had LGI-1-antibody-positive AE (two males and one female; M:F=2:1), with a mean age of 42.3 (±21.7). The LGI1-positive AE patient lost to follow-up. Comparing radiological lesions, MRS, and CASE scores from baseline to follow-up showed significant improvement (Table 7). All patients were in the remission phase (ARR=0).

**Table 7:** Efficacy of rituximab in a group with autoimmune encephalitis. NMDAR: N-methyl-D-aspartate receptor; CASE: Clinical Assessment Scale in AE; NA: not applicable.

Autoimmune Encephalitis (N=3)				
Age (in years)	42.3 ± 21.7			
Female; N (%)	1 (33.3%)			
GAD65 antibody-positive autoimmune encephalitis	1 (33.3%)			
NMDA-receptor antibody-positive autoimmune encephalitis	1 (33.3%)			
LGI-1 antibody-positive autoimmune encephalitis	1 (33.3%)			
Patients follow-up	At presentation (N=3)	At 6 months (N=2)	At 12 months (N=1)	At 18 months (N=1)
Radiological lesion	1.0 ± 1.0	NA	0	NA
Modified ranking scale	1.3 ± 1.2	0.0 ± 0.0	0	0
CASE score	3.3 ± 3.5	0.0 ± 0.0	0	0
Relapse episode/year	NA	NA	0	0

Patients with IMD

Two patients in the IMD group received treatment with rituximab, out of which one (50%) had anti-synthetase syndrome (anti-PL-7,12 positive), and one (50%) had dermatomyositis (anti-Mi-2 positive), (two females and 0 male; M:F=2:0) with a mean age of 51.0 (±17.0). Comparing CPK-NAC levels from baseline to follow-up showed significant improvement (Table 8). However, the patient with anti-synthetase syndrome (anti-PL-7,12 positive) experienced a relapse and subsequently switched to methotrexate, while the other patient with dermatomyositis responded well until the first maintenance dose. However, three months after the first maintenance dose, the patient developed Pott's spine. An interferon-gamma release assay (IGRA) test (TB-gold test) was done in this patient prior to starting RTX, resulting in the suspension of further subsequent doses of RTX.

**Table 8:** Efficacy of rituximab in a group with IMD. IMD: inflammatory disease; CPK-NAC: creatine phosphokinase-N-acetyl cysteine; NA: not applicable.

Inflammatory Muscle Disease (N=2)		
Age (in years)	51.0 ± 17.0	
Female; N (%)	2 (100%)	
Anti-synthetase syndrome	1 (50%)	
Dermatomyositis	1 (50%)	
Patients follow-up	At presentation (N=2)	At 6 months (N=2)
CPK-NAC level at presentation	1161.5 ± 1557.8	108.0 ± 82.0
Relapse episode/year	NA	1 (anti-synthetase syndrome)

Adverse drug reactions

In most patients, infusion-related reactions occurred during the first induction dose. These reactions were manageable by reducing the infusion rate. Afterward, patients tolerated RTX infusion well. Some patients experienced minor side effects, which were effectively managed. However, some patients experienced major side effects, such as one had cryptogenic organizing pneumonia. Another patient developed Pott's spine, leading to the suspension of subsequent RTX treatment at present (Table 9).

**Table 9:** Adverse drug reactions with rituximab administration.

Adverse Drug Reaction (ADR)	At 1st Dose (N=49)	At 2nd Dose (N=48)	At 6 Months (N=28)	At 12 Months (N=14)	At 18 Months (N=14)	At 24 Months (N=14)	At 30 Months (N=14)
Fever	06	00	00	00	00	00	00
Rash	07	00	01	00	00	00	00
Bradycardia	01	00	00	00	00	00	00
Tachycardia	03	01	00	00	00	02	00
Chills	13	00	00	00	00	00	00
Itching	01	00	00	00	00	00	00
Fungal infection	01	00	00	00	00	00	00
Acute hepatitis	02	00	00	00	00	00	00
Lower respiratory tract infection	00	00	00	01	00	00	00
Upper respiratory tract infection	03	00	00	00	00	00	00
Conjunctivitis	01	01	00	00	00	00	00
Joint pain	01	00	00	00	00	00	00
Back pain	01	00	00	00	00	00	00
Herpes zoster	00	00	00	01	00	00	00
Urinary tract infection	01	00	00	00	00	00	00
Headache	01	00	00	00	00	00	00
Pott's spine	00	00	01	00	00	00	00

## Discussion

The main objective of this study was to ascertain whether RTX is safe and effective in treating the following autoimmune diseases:

MG

In 1999, the first reported patient with refractory AChRA-positive MG showed significant improvement with RTX [13]. The BeatMG Study for AChRA-positive MG showed a mean age of 55.1 (±17.1), which was similar to our study. This study also demonstrated significant improvement in the MGFA class, similar to our study. The BeatMG trial provided Class I evidence [14]. The RINOMAX trial, a randomized controlled study, evaluated the effectiveness and safety of RTX in 87 participants with new-onset MG, encompassing AChRA-positive, MUSK-positive, and seronegative MG, with an average age of 67.4 (±13.4) years. The evaluation criteria included MGFA class, QMG score, MG-ADL, and MG-QoL. The results demonstrated statistically significant improvements in patients, indicating the efficacy of RTX in MG treatment, consistent with the findings from our study with an ARR of 0 [15]. In 2022, Inan et al. conducted a study on MUSK-positive MG, focusing on the MGFA class and ARR. This trial showed that standard immunosuppressive drugs were less effective against MUSK-positive MG patients, and these patients responded very well to RTX, similar to our study. Patients with MUSK-positive MG achieved remission [16]. Rivner et al. also demonstrated the effectiveness of RTX against anti-striatal muscle antibodies and LRP4 antibody-positive MG [17].

NMOSD

In May 2023, James et al. published a study conducted over two years at a tertiary care centre in South India, which closely resembled our study [18]. The study showed that the EDSS significantly decreased from 5.6 ± 2.5 to 3.3 ± 2.9, and there was a reduction in ARR, dropping from 0.5 ± 0.9 to 0.02 ± 0.08. Similarly, in our study, the EDSS decreased from 3.6 ± 1.3 to 1.5 ± 1.3 with an ARR of 0, and no serious side effects were seen in this group of patients [18]. In 2021, Uzunköprü et al. demonstrated the efficacy of RTX. The EDSS scores decreased from 3.94 ± 1.98 to 2.67 ± 1.54, and ARR decreased from 1.45 ± 1.53 to 0.15 ± 0.34. No radiological activity was observed or assessed at intervals of one year, which is similar to our study [19]. Both the RIN-1 study and the meta-analysis conducted by Gao et al. showcased promising results of RTX in this patient group [20,21].

MOGAD

In January 2023, Nepal et al. conducted a meta-analysis that demonstrated promising results for the off-label use of RTX for MOGAD, which included 13 studies and 238 patients. The analysis revealed that the ARR was lowered by 1.36 and EDSS by 0.52 [22]. In our study, there was a significant reduction in EDSS from 3.3 ± 0.4 to 1.4 ± 1.1, with an ARR of 0, and no new radiological activity was observed. Additionally, other studies by Bai et al. and Barreras et al. have demonstrated positive results in MOGAD patients with RTX treatment, similar to our study [23,24].

MS

In 2020, Ghajarzadeh et al. conducted a meta-analysis that demonstrated promising results for the off-label use of RTX for MS patients, which included 399 MS patients. The analysis revealed a reduction in EDSS by 0.29 and ARR by 1.24, with no new radiological activity detected. The most commonly reported adverse drug reaction was an infusion-related reaction, similar to our study. EDSS reduced from 3.1 ± 1.3 to 0.5 ± 0.9, and there was no radiological activity [25]. In 2008, the HERMES Trial led by Hauser et al. showcased a significant decrease in total gadolinium-enhancing lesions, mirroring findings in our study. As per protocol, we repeated MRI scans every year [26].

In 2021, Fatehi et al. showed substantial improvement with RTX in a patient with CIDP, which aligns with the findings in our study [11]. In 2018, a meta-analysis on vasculitic neuropathy conducted by Mena-Vázquez et al. demonstrated significant improvement in functional outcomes and disability, which aligns with our findings in cases of vasculitic neuropathy [12].

RTX also shows promising results in our study with the AE group, where improvement in MRS and CASE scores was observed, with an ARR of 0. Similar to the findings of Thaler et al. in their 2021 study and the meta-analysis published by Nepal et al. in 2020 [27,28].

In 2023, Gamba et al. studied the effectiveness of RTX in inflammatory autoimmune muscle diseases and demonstrated an excellent response, with improvement in CPK-NAC and clinical recovery, along with a significant reduction in ARR. However, in our study, one patient with anti-synthetase syndrome experienced a relapse, while another with dermatomyositis initially responded well to RTX therapy but later developed Pott's spine, leading to the hold of subsequent doses [29]. In 2022, Zhen et al. conducted a meta-analysis on idiopathic inflammatory myopathies (IIMs), presenting encouraging outcomes associated with RTX [30].

Our study had a few limitations. Firstly, it had a small sample size. Additionally, it was an observational study conducted at a single tertiary care centre. Furthermore, long-term follow-up is essential.

## Conclusions

This present study demonstrates excellent efficacy and a safe therapeutic option for immune-mediated neurological diseases with RTX. Compared to other immunomodulatory therapies, RTX has a generally good safety record. However, further long-term follow-up with a larger study group is necessary to ascertain extended long-term outcomes and establish guidelines for the duration of RTX treatment. This will help identify any potential long-term complications associated with RTX therapy.

## Figures and Tables

**Figure 5:**
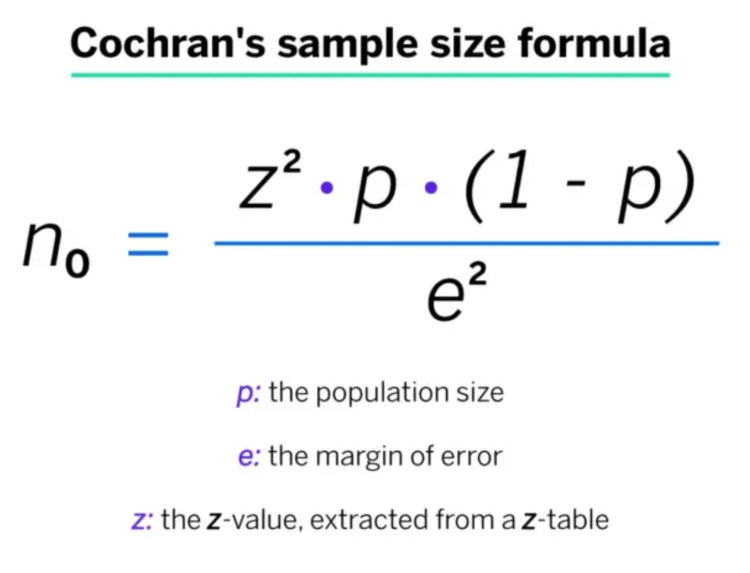
The formula used for the sample size calculation. p=0.04, e=0.08, and z=1.96. Reference study: https://www.ncbi.nlm.nih.gov/pmc/articles/PMC8292678/

## Appendices

Sample size calculation

Based on a previous study referred to below, with a 95% confidence interval, there is a clinically meaningful difference of 8%, and the prevalence of autoimmune neurologic disorders is 4%. Accounting for a 10% non-inclusion or drop-out rate, the minimum required sample size should be 28 participants. After rounding off, the finalized sample size was 30 participants.
